# Crystal structure of *fac*-[2-(4-methyl-5-phenyl­pyridin-2-yl)phenyl-κ^2^ *C* ^1^,*N*]bis­[2-(pyridin-2-yl)phenyl-κ^2^ *C* ^1^,*N*]iridium(III)

**DOI:** 10.1107/S2056989016017618

**Published:** 2016-11-08

**Authors:** Chi-Heon Lee, Suk-Hee Moon, Ki-Min Park, Youngjin Kang

**Affiliations:** a Division of Science Education, Kangwon National University, Chuncheon 24341, Republic of Korea; b Department of Food and Nutrition, Kyungnam College of Information and Technology, Busan 47011, Republic of Korea; c Research Institute of Natural Science, Gyeongsang National University, Jinju, 52828, Republic of Korea

**Keywords:** crystal structure, iridium(III) complex, *C*,*N*-bidentate ligand, *fac*-C_3_N_3_ coordination set, π–π stacking inter­actions

## Abstract

In the title compound, [Ir(C_11_H_8_N)_2_(C_18_H_14_N)], the Ir^III^ ion adopts a distorted octa­hedral coordination environment defined by three *C*,*N*-chelating ligands, one stemming from a 2-(4-phenyl-5-methyl­pyridin-2-yl)phenyl ligand and two from 2-(pyridin-2-yl)phenyl ligands, arranged in a facial manner. The Ir^III^ ion lies almost in the equatorial plane [deviation = 0.0069 (15) Å]. In the crystal, inter­molecular π–π stacking inter­actions, as well as inter­molecular C—H⋯π inter­actions, are present, leading to a three-dimensional network.

## Abstract 2

The Ir^III^ atom in the title mol­ecule adopts a distorted octa­hedral C_3_N_3_ coordination environment, being *C*,*N*-chelated by two 2-(pyridin-2-yl)phenyl ligands and one 2-(4-phenyl-5-methyl­pyridin-2-yl)phenyl ligand.

## Chemical context   

Cyclo­metallated iridium(III) complexes with the chelating ligand 2-phenyl­pyridine (C
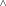
N) are of great inter­est in phospho­rescence organic light-emitting diodes (OLEDs) due to their high quantum efficiency and easy tuning emission energy (Kang *et al.*, 2013 ▸). In general, iridium(III) complexes with chelating C
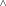
N ligands can be divided into two groups, homoleptic and heteroleptic complexes, according to the coordination environment of the central Ir^III^ atom. The structural characteristics involving other chemical/electronic properties for both homoleptic Ir(C
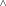
N)_3_ and heteroleptic Ir(C
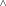
N)_2_(*L* 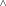 *X*) complexes, where *L* 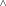 *X* is a monoanionic O
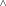
O or N
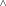
O ligand, have been well explored over the past two decades (Chi & Chou, 2010 ▸). However, reports of the mol­ecular and crystal structures of heteroleptic Ir^III^ compounds with the same chelating modes, *viz.* Ir(C
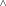
N)_2_(C
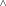
N)′, are very scarce compared to those for Ir(C
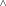
N)_2_(*L* 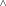 *X*) (Jung *et al.*, 2012 ▸; Natori *et al.*, 2013 ▸). Herein, we describe the structure of the title Ir^III^ complex, *fac*-{2-[(4-phenyl-5-meth­yl)pyridine-2-yl]phenyl-*κ* ^2^ *C* ^1^,*N*}bis­[2-(pyridine-2-yl)phenyl-*κ* ^2^ *C* ^1^,*N*]iridium(III), which was synthesized by the reaction of [(C
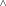
N)_2_Ir(*μ*-Cl)]_2_ and 4-methyl-2,5-di­phenyl­pyridine in the presence of Ag^I^.

## Structural commentary   

**Figure 1:**
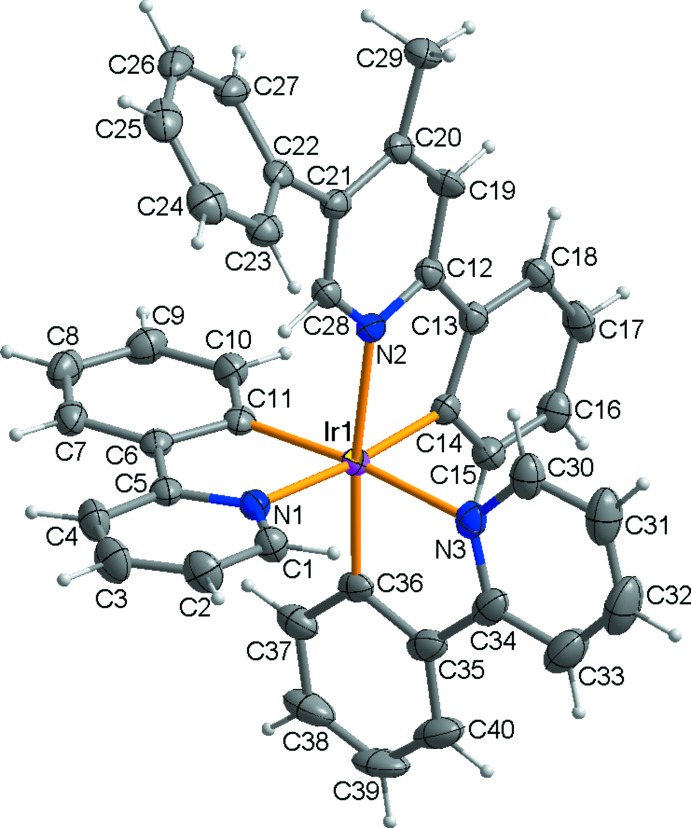
View of the mol­ecular structure of the title compound, showing the atom-numbering scheme. Displacement ellipsoids are drawn at the 50% probability level.

In the title compound, the asymmetric unit comprises of one Ir^III^ ion, two 2-phenyl­pyridine ligands, and one 4-methyl-2,5-di­phenyl­pyridine ligand (Fig. 1 ▸). The Ir^III^ ion is six-coordin­ated by the three *C*,*N*-bidentate ligands, giving rise to a distorted octa­hedral coordination environment with bond angles falling in the range 79.27 (12) to 97.37 (13)°. As shown in Table 1 ▸, the Ir—C and Ir—N bond lengths in the title compound are within the ranges reported for similar Ir^III^ compounds (Jung *et al.*, 2012 ▸). The pyridyl N atoms of the three ligands are arranged in a *fac*-configuration around the octa­hedrally coordinated Ir^III^ ion. The equatorial plane is defined by the N1/N3/C14/C11 atoms, the mean deviation from the least-squares plane being 0.081 Å. The Ir^III^ ion lies almost in the equatorial plane with a deviation of 0.0069 (15) Å. Within the 2-(pyridine-2-yl)phenyl ligands, the dihedral angles between the aromatic rings are 5.6 (2) (between rings N1/C1–C5 and C6–C11) and 5.9 (2)° (between rings N3/C30–C34 and C35–C40). Within the 2-[(4-phenyl-5-meth­yl)pyridine-2-yl]phenyl ligand, the dihedral angles between the central pyridine ring and the phenyl rings at either end are 1.3 (2) and 43.84 (12)° for the C13–C18 and C22–C27 rings, respectively.
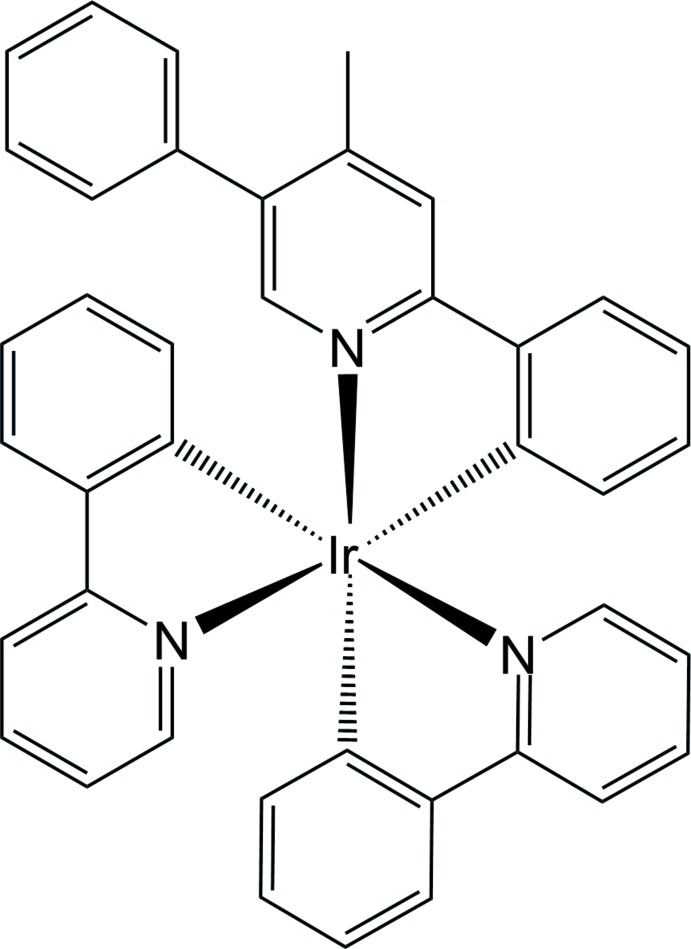


## Supra­molecular features   

**Figure 2:**
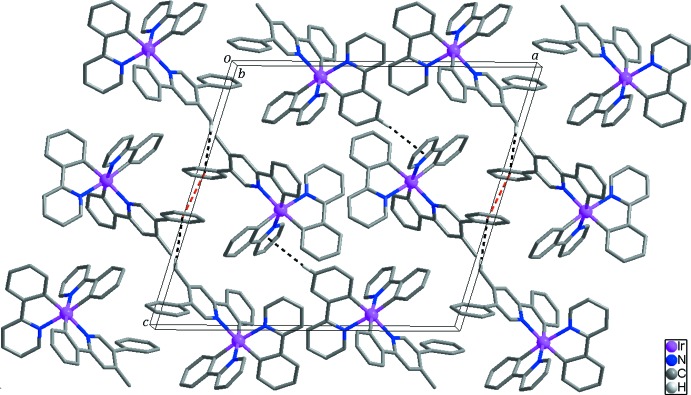
Packing plot of the mol­ecular components in the title compound. Red and black dashed lines represent inter­molecular π–π stacking inter­actions and C—H⋯π inter­actions, respectively. H atoms not involved in inter­molecular inter­actions have been omitted for clarity.

Inter­molecular π–π stacking inter­actions [*Cg*1⋯*Cg*1^i^ = 3.838 (2) Å; *Cg*1 is the centroid of the C22–C27 ring; symmetry code: (i) −*x*, −*y* + 2, −*z*] occur in the crystal structure of the title compound (Fig. 2 ▸). In addition, weak inter­molecular C—H⋯π inter­actions (Table 2 ▸) contribute to the stabilization of the crystal structure.

## Synthesis and crystallization   

The ligand 4-methyl-2,5-di­phenyl­pyridine was synthesized according to a literature procedure (Zhou *et al.*, 2013 ▸). The title Ir^III^ complex was also prepared according to a literature protocol (Jung *et al.*, 2012 ▸). Crystals of the title complex were obtained by allowing a di­chloro­methane/hexane solution to evaporate slowly at room temperature.

## Refinement   

Crystal data, data collection and structure refinement details are summarized in Table 3 ▸. A reflection affected by the beamstop (100) was omitted from the final refinement. All H atoms were positioned geometrically and refined using a riding model, with *d*(C—H) = 0.95 Å for C*sp* ^2^–H, and 0.98 Å for methyl H atoms. For all H atoms, *U*_iso_(H) = 1.2*U*_eq_ of the parent atom.

## Supplementary Material

Crystal structure: contains datablock(s) I, New_Global_Publ_Block. DOI: 10.1107/S2056989016017618/wm5336sup1.cif

Structure factors: contains datablock(s) I. DOI: 10.1107/S2056989016017618/wm5336Isup2.hkl

CCDC reference: 1515004

Additional supporting information: crystallographic information; 3D view; checkCIF report

## Figures and Tables

**Table 1:** Selected geometric parameters (Å, °)

Ir1—C14	2.006 (3)	Ir1—N1	2.117 (3)
Ir1—C36	2.010 (3)	Ir1—N2	2.122 (3)
Ir1—C11	2.010 (3)	Ir1—N3	2.125 (3)
			
C14—Ir1—C36	94.78 (13)	C11—Ir1—N2	88.63 (11)
C14—Ir1—C11	97.37 (13)	N1—Ir1—N2	96.33 (11)
C36—Ir1—C11	95.40 (13)	C14—Ir1—N3	86.75 (11)
C14—Ir1—N1	174.67 (11)	C36—Ir1—N3	79.51 (13)
C36—Ir1—N1	89.78 (12)	C11—Ir1—N3	173.73 (12)
C11—Ir1—N1	79.41 (12)	N1—Ir1—N3	96.81 (10)
C14—Ir1—N2	79.27 (12)	N2—Ir1—N3	96.80 (11)
C36—Ir1—N2	173.22 (11)		

**Table 2:** Hydrogen-bond geometry (Å, °) *Cg*1 and *Cg*2 are the centroids of the C22–C27 and N1/C1–C5 rings, respectively.

*D*—H⋯*A*	*D*—H	H⋯*A*	*D*⋯*A*	*D*—H⋯*A*
C29—H29*A*⋯*Cg*1^i^	0.98	2.89	3.589 (4)	136
C39—H39⋯*Cg*2^ii^	0.95	2.89	3.796 (5)	160

Symmetry codes: (i) 
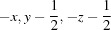
; (ii) 
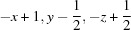
.

**Table 3:** Experimental details

Crystal data	
Chemical formula	[Ir(C_11_H_8_N)_2_(C_18_H_14_N)]
*M*_r_	744.87
Crystal system, space group	Monoclinic, *P*2_1_/*c*
Temperature (K)	173
*a*, *b*, *c* (Å)	19.8293 (3), 8.6464 (1), 18.1551 (3)
β (°)	106.715 (1)
*V* (Å^3^)	2981.21 (8)
*Z*	4
Radiation type	Mo *K*α
μ (mm^−1^)	4.51
Crystal size (mm)	0.30 × 0.25 × 0.17

Data collection	
Diffractometer	Bruker APEXII CCD
Absorption correction	Multi-scan (*SADABS*; Bruker, 2013 ▸)
*T*_min_, *T*_max_	0.521, 0.746
No. of measured, independent and observed [*I* > 2σ(*I*)] reflections	27408, 6855, 6080
*R*_int_	0.033
(sin θ/λ)_max_ (Å^−1^)	0.651

Refinement	
*R*[*F* ^2^ > 2σ(*F* ^2^)], *wR*(*F* ^2^), *S*	0.026, 0.062, 1.02
No. of reflections	6855
No. of parameters	397
H-atom treatment	H-atom parameters constrained
Δρ_max_, Δρ_min_ (e Å^−3^)	1.77, −0.71

Computer programs: *APEX2* and *SAINT* (Bruker, 2013 ▸), *SHELXS97* and *SHELXTL* (Sheldrick, 2008 ▸), *SHELXL2014* (Sheldrick, 2015 ▸) and *DIAMOND* (Brandenburg, 2010 ▸).

## supplementary crystallographic information

### Crystal data

**Table:**

[Ir(C_11_H_8_N)_2_(C_18_H_14_N)]	*F*(000) = 1472
*M**_r_* = 744.87	*D*_x_ = 1.660 Mg m^−^^3^
Monoclinic, *P*2_1_/*c*	Mo *K*α radiation, λ = 0.71073 Å
*a* = 19.8293 (3) Å	Cell parameters from 9925 reflections
*b* = 8.6464 (1) Å	θ = 2.3–27.5°
*c* = 18.1551 (3) Å	µ = 4.51 mm^−^^1^
β = 106.715 (1)°	*T* = 173 K
*V* = 2981.21 (8) Å^3^	Block, yellow
*Z* = 4	0.30 × 0.25 × 0.17 mm

### Data collection

**Table:**

Bruker APEXII CCD diffractometer	6080 reflections with *I* > 2σ(*I*)
φ and ω scans	*R*_int_ = 0.033
Absorption correction: multi-scan (*SADABS*; Bruker, 2013)	θ_max_ = 27.6°, θ_min_ = 2.2°
*T*_min_ = 0.521, *T*_max_ = 0.746	*h* = −25→25
27408 measured reflections	*k* = −11→11
6855 independent reflections	*l* = −23→23

### Refinement

**Table:**

Refinement on *F*^2^	0 restraints
Least-squares matrix: full	Hydrogen site location: inferred from neighbouring sites
*R*[*F*^2^ > 2σ(*F*^2^)] = 0.026	H-atom parameters constrained
*wR*(*F*^2^) = 0.062	*w* = 1/[σ^2^(*F*_o_^2^) + (0.0283*P*)^2^ + 4.5659*P*] where *P* = (*F*_o_^2^ + 2*F*_c_^2^)/3
*S* = 1.02	(Δ/σ)_max_ = 0.001
6855 reflections	Δρ_max_ = 1.77 e Å^−^^3^
397 parameters	Δρ_min_ = −0.71 e Å^−^^3^

### Special details

**Table:**

Geometry. All esds (except the esd in the dihedral angle between two l.s. planes) are estimated using the full covariance matrix. The cell esds are taken into account individually in the estimation of esds in distances, angles and torsion angles; correlations between esds in cell parameters are only used when they are defined by crystal symmetry. An approximate (isotropic) treatment of cell esds is used for estimating esds involving l.s. planes.

### Fractional atomic coordinates and isotropic or equivalent isotropic displacement parameters (Å^2^)

**Table:**

	*x*	*y*	*z*	*U*_iso_*/*U*_eq_	
Ir1	0.29220 (2)	0.64276 (2)	0.04767 (2)	0.02005 (5)	
N1	0.29261 (14)	0.8427 (3)	0.11505 (15)	0.0229 (6)	
N2	0.19761 (13)	0.6951 (3)	−0.03909 (15)	0.0221 (5)	
N3	0.36253 (15)	0.7274 (3)	−0.01184 (16)	0.0278 (6)	
C1	0.32224 (19)	0.9782 (4)	0.10758 (19)	0.0292 (7)	
H1	0.3448	0.9885	0.0682	0.035*	
C2	0.3217 (2)	1.1028 (4)	0.1537 (2)	0.0364 (8)	
H2	0.3427	1.1979	0.1459	0.044*	
C3	0.2901 (2)	1.0878 (5)	0.2118 (2)	0.0390 (9)	
H3	0.2896	1.1721	0.2452	0.047*	
C4	0.2595 (2)	0.9492 (4)	0.2208 (2)	0.0343 (8)	
H4	0.2379	0.9371	0.2608	0.041*	
C5	0.25996 (17)	0.8260 (4)	0.17116 (19)	0.0246 (7)	
C6	0.22626 (17)	0.6753 (4)	0.17108 (19)	0.0241 (7)	
C7	0.18657 (19)	0.6405 (4)	0.2211 (2)	0.0301 (8)	
H7	0.1826	0.7142	0.2585	0.036*	
C8	0.15301 (19)	0.4997 (5)	0.2166 (2)	0.0350 (8)	
H8	0.1257	0.4762	0.2505	0.042*	
C9	0.1596 (2)	0.3924 (4)	0.1620 (2)	0.0342 (8)	
H9	0.1371	0.2947	0.1589	0.041*	
C10	0.19868 (18)	0.4272 (4)	0.1123 (2)	0.0299 (7)	
H10	0.2020	0.3525	0.0751	0.036*	
C11	0.23371 (16)	0.5691 (4)	0.11465 (17)	0.0231 (6)	
C12	0.17645 (17)	0.5849 (4)	−0.09425 (18)	0.0244 (7)	
C13	0.22309 (17)	0.4510 (4)	−0.08479 (18)	0.0233 (6)	
C14	0.28203 (16)	0.4536 (4)	−0.01860 (18)	0.0233 (7)	
C15	0.32853 (18)	0.3276 (4)	−0.0099 (2)	0.0275 (7)	
H15	0.3689	0.3247	0.0336	0.033*	
C16	0.31741 (19)	0.2077 (4)	−0.0626 (2)	0.0310 (8)	
H16	0.3504	0.1253	−0.0550	0.037*	
C17	0.2591 (2)	0.2069 (4)	−0.1257 (2)	0.0312 (8)	
H17	0.2515	0.1237	−0.1613	0.037*	
C18	0.21153 (19)	0.3280 (4)	−0.13697 (19)	0.0280 (7)	
H18	0.1709	0.3276	−0.1803	0.034*	
C19	0.11442 (18)	0.6060 (4)	−0.1549 (2)	0.0294 (8)	
H19	0.1004	0.5301	−0.1942	0.035*	
C20	0.07314 (17)	0.7395 (4)	−0.15741 (18)	0.0259 (7)	
C21	0.09467 (17)	0.8491 (4)	−0.09800 (19)	0.0239 (7)	
C22	0.05436 (17)	0.9901 (4)	−0.09133 (17)	0.0249 (7)	
C23	0.08800 (19)	1.1305 (4)	−0.0697 (2)	0.0306 (8)	
H23	0.1374	1.1375	−0.0614	0.037*	
C24	0.0506 (2)	1.2615 (5)	−0.0599 (2)	0.0373 (9)	
H24	0.0746	1.3565	−0.0448	0.045*	
C25	−0.0211 (2)	1.2533 (5)	−0.0722 (2)	0.0394 (9)	
H25	−0.0468	1.3428	−0.0661	0.047*	
C26	−0.0557 (2)	1.1141 (5)	−0.0935 (2)	0.0367 (9)	
H26	−0.1052	1.1082	−0.1022	0.044*	
C27	−0.01834 (18)	0.9837 (5)	−0.10215 (19)	0.0305 (8)	
H27	−0.0424	0.8882	−0.1156	0.037*	
C28	0.15737 (17)	0.8197 (4)	−0.04181 (18)	0.0234 (7)	
H28	0.1729	0.8943	−0.0021	0.028*	
C29	0.00979 (19)	0.7602 (5)	−0.2259 (2)	0.0345 (8)	
H29A	0.0048	0.6701	−0.2597	0.041*	
H29B	−0.0324	0.7707	−0.2085	0.041*	
H29C	0.0157	0.8535	−0.2540	0.041*	
C30	0.3439 (2)	0.7941 (4)	−0.0815 (2)	0.0360 (8)	
H30	0.2953	0.8109	−0.1062	0.043*	
C31	0.3921 (3)	0.8394 (5)	−0.1188 (3)	0.0489 (11)	
H31	0.3773	0.8862	−0.1682	0.059*	
C32	0.4622 (3)	0.8150 (6)	−0.0825 (3)	0.0596 (14)	
H32	0.4968	0.8455	−0.1066	0.072*	
C33	0.4820 (2)	0.7464 (6)	−0.0116 (3)	0.0534 (12)	
H33	0.5305	0.7304	0.0138	0.064*	
C34	0.43158 (19)	0.7001 (4)	0.0236 (2)	0.0338 (8)	
C35	0.44516 (19)	0.6172 (4)	0.0971 (2)	0.0337 (8)	
C36	0.38503 (17)	0.5785 (4)	0.12033 (19)	0.0261 (7)	
C37	0.3974 (2)	0.4950 (4)	0.1889 (2)	0.0350 (8)	
H37	0.3584	0.4691	0.2072	0.042*	
C38	0.4639 (2)	0.4488 (5)	0.2311 (2)	0.0471 (11)	
H38	0.4701	0.3916	0.2772	0.057*	
C39	0.5216 (2)	0.4860 (6)	0.2059 (3)	0.0525 (12)	
H39	0.5674	0.4527	0.2344	0.063*	
C40	0.5128 (2)	0.5708 (5)	0.1401 (3)	0.0483 (11)	
H40	0.5526	0.5982	0.1236	0.058*	

### Atomic displacement parameters (Å^2^)

**Table:**

	*U*^11^	*U*^22^	*U*^33^	*U*^12^	*U*^13^	*U*^23^
Ir1	0.01823 (7)	0.02267 (7)	0.01843 (7)	0.00119 (5)	0.00394 (5)	−0.00203 (5)
N1	0.0223 (14)	0.0243 (14)	0.0211 (13)	0.0011 (11)	0.0049 (11)	−0.0029 (11)
N2	0.0186 (13)	0.0271 (14)	0.0206 (13)	−0.0020 (11)	0.0055 (11)	0.0004 (11)
N3	0.0306 (15)	0.0247 (15)	0.0309 (15)	−0.0012 (12)	0.0137 (13)	−0.0080 (12)
C1	0.0325 (18)	0.0304 (18)	0.0254 (17)	−0.0015 (15)	0.0093 (15)	−0.0016 (14)
C2	0.046 (2)	0.0267 (18)	0.037 (2)	−0.0028 (16)	0.0114 (18)	−0.0050 (15)
C3	0.050 (2)	0.032 (2)	0.036 (2)	0.0037 (18)	0.0148 (18)	−0.0109 (16)
C4	0.042 (2)	0.036 (2)	0.0280 (18)	0.0034 (17)	0.0148 (16)	−0.0065 (15)
C5	0.0205 (16)	0.0303 (18)	0.0212 (16)	0.0049 (13)	0.0032 (13)	−0.0002 (13)
C6	0.0200 (16)	0.0286 (17)	0.0225 (16)	0.0055 (13)	0.0041 (13)	0.0016 (13)
C7	0.0276 (18)	0.038 (2)	0.0260 (17)	0.0079 (15)	0.0090 (14)	0.0017 (14)
C8	0.0306 (19)	0.045 (2)	0.0321 (19)	0.0012 (17)	0.0142 (16)	0.0092 (16)
C9	0.0301 (19)	0.033 (2)	0.040 (2)	−0.0039 (15)	0.0112 (16)	0.0051 (16)
C10	0.0278 (17)	0.0333 (19)	0.0283 (17)	0.0003 (15)	0.0076 (14)	−0.0035 (15)
C11	0.0201 (15)	0.0285 (17)	0.0193 (15)	0.0060 (14)	0.0034 (12)	0.0048 (13)
C12	0.0264 (16)	0.0287 (17)	0.0194 (15)	−0.0038 (14)	0.0084 (13)	0.0006 (13)
C13	0.0246 (16)	0.0254 (17)	0.0211 (15)	−0.0030 (13)	0.0084 (13)	−0.0005 (12)
C14	0.0214 (15)	0.0269 (17)	0.0233 (16)	−0.0027 (13)	0.0093 (13)	−0.0009 (13)
C15	0.0264 (17)	0.0321 (19)	0.0236 (17)	0.0010 (14)	0.0068 (14)	−0.0015 (13)
C16	0.037 (2)	0.0231 (17)	0.036 (2)	0.0037 (15)	0.0158 (16)	−0.0016 (15)
C17	0.043 (2)	0.0247 (17)	0.0277 (18)	−0.0050 (16)	0.0133 (16)	−0.0093 (14)
C18	0.0310 (18)	0.0303 (18)	0.0228 (17)	−0.0074 (14)	0.0080 (14)	−0.0036 (13)
C19	0.0266 (18)	0.0332 (19)	0.0254 (17)	−0.0091 (14)	0.0027 (14)	−0.0098 (14)
C20	0.0232 (16)	0.0369 (19)	0.0178 (15)	−0.0037 (14)	0.0063 (13)	0.0024 (13)
C21	0.0198 (15)	0.0308 (18)	0.0214 (16)	−0.0021 (13)	0.0063 (13)	0.0040 (13)
C22	0.0209 (16)	0.0358 (19)	0.0167 (15)	0.0039 (14)	0.0034 (12)	0.0048 (13)
C23	0.0271 (18)	0.036 (2)	0.0272 (18)	0.0018 (15)	0.0056 (15)	0.0054 (14)
C24	0.045 (2)	0.035 (2)	0.031 (2)	0.0041 (17)	0.0082 (17)	0.0058 (15)
C25	0.044 (2)	0.043 (2)	0.031 (2)	0.0192 (19)	0.0109 (17)	0.0076 (17)
C26	0.0248 (18)	0.058 (3)	0.0265 (18)	0.0140 (17)	0.0066 (15)	0.0092 (17)
C27	0.0216 (17)	0.043 (2)	0.0251 (17)	0.0008 (15)	0.0045 (14)	0.0063 (15)
C28	0.0242 (16)	0.0267 (17)	0.0190 (15)	−0.0016 (13)	0.0057 (13)	−0.0016 (12)
C29	0.0263 (18)	0.049 (2)	0.0240 (18)	0.0034 (16)	0.0005 (14)	−0.0027 (15)
C30	0.046 (2)	0.0297 (19)	0.038 (2)	−0.0017 (17)	0.0203 (18)	−0.0042 (16)
C31	0.069 (3)	0.038 (2)	0.053 (3)	−0.006 (2)	0.039 (3)	−0.0018 (19)
C32	0.062 (3)	0.059 (3)	0.077 (4)	−0.012 (2)	0.051 (3)	−0.006 (3)
C33	0.034 (2)	0.061 (3)	0.072 (3)	−0.007 (2)	0.026 (2)	−0.012 (2)
C34	0.0279 (18)	0.0304 (19)	0.046 (2)	−0.0045 (15)	0.0156 (17)	−0.0129 (17)
C35	0.0236 (17)	0.034 (2)	0.041 (2)	0.0006 (14)	0.0042 (16)	−0.0131 (15)
C36	0.0227 (16)	0.0233 (16)	0.0291 (17)	0.0023 (14)	0.0021 (13)	−0.0104 (14)
C37	0.036 (2)	0.034 (2)	0.0280 (19)	0.0060 (16)	−0.0020 (15)	−0.0055 (15)
C38	0.045 (2)	0.045 (2)	0.036 (2)	0.011 (2)	−0.0133 (18)	−0.0080 (18)
C39	0.029 (2)	0.056 (3)	0.054 (3)	0.012 (2)	−0.0163 (19)	−0.012 (2)
C40	0.0219 (19)	0.054 (3)	0.063 (3)	0.0017 (19)	0.0020 (18)	−0.017 (2)

### Geometric parameters (Å, º)

**Table:**

Ir1—C14	2.006 (3)	C18—H18	0.9500
Ir1—C36	2.010 (3)	C19—C20	1.408 (5)
Ir1—C11	2.010 (3)	C19—H19	0.9500
Ir1—N1	2.117 (3)	C20—C21	1.407 (5)
Ir1—N2	2.122 (3)	C20—C29	1.502 (5)
Ir1—N3	2.125 (3)	C21—C28	1.386 (5)
N1—C1	1.335 (4)	C21—C22	1.482 (5)
N1—C5	1.363 (4)	C22—C23	1.386 (5)
N2—C28	1.333 (4)	C22—C27	1.399 (4)
N2—C12	1.359 (4)	C23—C24	1.393 (5)
N3—C30	1.342 (5)	C23—H23	0.9500
N3—C34	1.356 (5)	C24—C25	1.376 (5)
C1—C2	1.367 (5)	C24—H24	0.9500
C1—H1	0.9500	C25—C26	1.383 (6)
C2—C3	1.379 (5)	C25—H25	0.9500
C2—H2	0.9500	C26—C27	1.382 (5)
C3—C4	1.375 (5)	C26—H26	0.9500
C3—H3	0.9500	C27—H27	0.9500
C4—C5	1.396 (5)	C28—H28	0.9500
C4—H4	0.9500	C29—H29A	0.9800
C5—C6	1.465 (5)	C29—H29B	0.9800
C6—C7	1.395 (5)	C29—H29C	0.9800
C6—C11	1.415 (5)	C30—C31	1.377 (5)
C7—C8	1.379 (5)	C30—H30	0.9500
C7—H7	0.9500	C31—C32	1.372 (7)
C8—C9	1.390 (5)	C31—H31	0.9500
C8—H8	0.9500	C32—C33	1.369 (7)
C9—C10	1.383 (5)	C32—H32	0.9500
C9—H9	0.9500	C33—C34	1.391 (5)
C10—C11	1.404 (5)	C33—H33	0.9500
C10—H10	0.9500	C34—C35	1.469 (6)
C12—C19	1.407 (5)	C35—C40	1.403 (5)
C12—C13	1.460 (5)	C35—C36	1.415 (5)
C13—C18	1.399 (4)	C36—C37	1.399 (5)
C13—C14	1.415 (4)	C37—C38	1.379 (5)
C14—C15	1.406 (5)	C37—H37	0.9500
C15—C16	1.385 (5)	C38—C39	1.388 (7)
C15—H15	0.9500	C38—H38	0.9500
C16—C17	1.374 (5)	C39—C40	1.369 (7)
C16—H16	0.9500	C39—H39	0.9500
C17—C18	1.385 (5)	C40—H40	0.9500
C17—H17	0.9500		
			
C14—Ir1—C36	94.78 (13)	C18—C17—H17	120.2
C14—Ir1—C11	97.37 (13)	C17—C18—C13	120.2 (3)
C36—Ir1—C11	95.40 (13)	C17—C18—H18	119.9
C14—Ir1—N1	174.67 (11)	C13—C18—H18	119.9
C36—Ir1—N1	89.78 (12)	C12—C19—C20	120.1 (3)
C11—Ir1—N1	79.41 (12)	C12—C19—H19	120.0
C14—Ir1—N2	79.27 (12)	C20—C19—H19	120.0
C36—Ir1—N2	173.22 (11)	C21—C20—C19	118.8 (3)
C11—Ir1—N2	88.63 (11)	C21—C20—C29	123.4 (3)
N1—Ir1—N2	96.33 (11)	C19—C20—C29	117.7 (3)
C14—Ir1—N3	86.75 (11)	C28—C21—C20	116.8 (3)
C36—Ir1—N3	79.51 (13)	C28—C21—C22	118.8 (3)
C11—Ir1—N3	173.73 (12)	C20—C21—C22	124.4 (3)
N1—Ir1—N3	96.81 (10)	C23—C22—C27	117.8 (3)
N2—Ir1—N3	96.80 (11)	C23—C22—C21	121.1 (3)
C1—N1—C5	119.1 (3)	C27—C22—C21	120.9 (3)
C1—N1—Ir1	125.9 (2)	C22—C23—C24	121.2 (3)
C5—N1—Ir1	115.0 (2)	C22—C23—H23	119.4
C28—N2—C12	119.0 (3)	C24—C23—H23	119.4
C28—N2—Ir1	126.3 (2)	C25—C24—C23	120.1 (4)
C12—N2—Ir1	114.6 (2)	C25—C24—H24	120.0
C30—N3—C34	119.3 (3)	C23—C24—H24	120.0
C30—N3—Ir1	125.8 (2)	C24—C25—C26	119.7 (4)
C34—N3—Ir1	114.8 (2)	C24—C25—H25	120.2
N1—C1—C2	123.3 (3)	C26—C25—H25	120.2
N1—C1—H1	118.4	C27—C26—C25	120.2 (3)
C2—C1—H1	118.4	C27—C26—H26	119.9
C1—C2—C3	118.7 (4)	C25—C26—H26	119.9
C1—C2—H2	120.7	C26—C27—C22	121.0 (4)
C3—C2—H2	120.7	C26—C27—H27	119.5
C4—C3—C2	119.1 (3)	C22—C27—H27	119.5
C4—C3—H3	120.5	N2—C28—C21	125.2 (3)
C2—C3—H3	120.5	N2—C28—H28	117.4
C3—C4—C5	120.2 (3)	C21—C28—H28	117.4
C3—C4—H4	119.9	C20—C29—H29A	109.5
C5—C4—H4	119.9	C20—C29—H29B	109.5
N1—C5—C4	119.6 (3)	H29A—C29—H29B	109.5
N1—C5—C6	114.1 (3)	C20—C29—H29C	109.5
C4—C5—C6	126.3 (3)	H29A—C29—H29C	109.5
C7—C6—C11	121.9 (3)	H29B—C29—H29C	109.5
C7—C6—C5	122.2 (3)	N3—C30—C31	122.9 (4)
C11—C6—C5	115.8 (3)	N3—C30—H30	118.5
C8—C7—C6	120.3 (3)	C31—C30—H30	118.5
C8—C7—H7	119.9	C32—C31—C30	118.1 (4)
C6—C7—H7	119.9	C32—C31—H31	120.9
C7—C8—C9	119.3 (3)	C30—C31—H31	120.9
C7—C8—H8	120.4	C33—C32—C31	119.7 (4)
C9—C8—H8	120.4	C33—C32—H32	120.2
C10—C9—C8	120.4 (3)	C31—C32—H32	120.2
C10—C9—H9	119.8	C32—C33—C34	120.5 (4)
C8—C9—H9	119.8	C32—C33—H33	119.8
C9—C10—C11	122.4 (3)	C34—C33—H33	119.8
C9—C10—H10	118.8	N3—C34—C33	119.5 (4)
C11—C10—H10	118.8	N3—C34—C35	114.3 (3)
C10—C11—C6	115.8 (3)	C33—C34—C35	126.1 (4)
C10—C11—Ir1	128.7 (2)	C40—C35—C36	121.3 (4)
C6—C11—Ir1	115.5 (2)	C40—C35—C34	122.8 (4)
N2—C12—C19	120.0 (3)	C36—C35—C34	115.9 (3)
N2—C12—C13	114.7 (3)	C37—C36—C35	116.2 (3)
C19—C12—C13	125.2 (3)	C37—C36—Ir1	128.3 (3)
C18—C13—C14	121.4 (3)	C35—C36—Ir1	115.5 (3)
C18—C13—C12	123.2 (3)	C38—C37—C36	122.5 (4)
C14—C13—C12	115.3 (3)	C38—C37—H37	118.8
C15—C14—C13	116.0 (3)	C36—C37—H37	118.8
C15—C14—Ir1	127.9 (2)	C37—C38—C39	119.9 (4)
C13—C14—Ir1	116.0 (2)	C37—C38—H38	120.0
C16—C15—C14	122.2 (3)	C39—C38—H38	120.0
C16—C15—H15	118.9	C40—C39—C38	120.1 (4)
C14—C15—H15	118.9	C40—C39—H39	120.0
C17—C16—C15	120.6 (3)	C38—C39—H39	120.0
C17—C16—H16	119.7	C39—C40—C35	120.1 (4)
C15—C16—H16	119.7	C39—C40—H40	120.0
C16—C17—C18	119.6 (3)	C35—C40—H40	120.0
C16—C17—H17	120.2		
			
C5—N1—C1—C2	0.0 (5)	C12—C19—C20—C29	176.5 (3)
Ir1—N1—C1—C2	−179.0 (3)	C19—C20—C21—C28	2.3 (4)
N1—C1—C2—C3	1.2 (6)	C29—C20—C21—C28	−174.9 (3)
C1—C2—C3—C4	−0.9 (6)	C19—C20—C21—C22	−176.6 (3)
C2—C3—C4—C5	−0.5 (6)	C29—C20—C21—C22	6.2 (5)
C1—N1—C5—C4	−1.5 (5)	C28—C21—C22—C23	42.5 (4)
Ir1—N1—C5—C4	177.6 (3)	C20—C21—C22—C23	−138.7 (3)
C1—N1—C5—C6	176.9 (3)	C28—C21—C22—C27	−133.6 (3)
Ir1—N1—C5—C6	−4.0 (4)	C20—C21—C22—C27	45.3 (4)
C3—C4—C5—N1	1.7 (5)	C27—C22—C23—C24	−0.8 (5)
C3—C4—C5—C6	−176.5 (4)	C21—C22—C23—C24	−177.0 (3)
N1—C5—C6—C7	−175.9 (3)	C22—C23—C24—C25	−0.4 (5)
C4—C5—C6—C7	2.4 (5)	C23—C24—C25—C26	0.7 (5)
N1—C5—C6—C11	1.3 (4)	C24—C25—C26—C27	0.2 (5)
C4—C5—C6—C11	179.6 (3)	C25—C26—C27—C22	−1.4 (5)
C11—C6—C7—C8	−0.1 (5)	C23—C22—C27—C26	1.7 (5)
C5—C6—C7—C8	176.9 (3)	C21—C22—C27—C26	177.9 (3)
C6—C7—C8—C9	0.4 (5)	C12—N2—C28—C21	−1.1 (5)
C7—C8—C9—C10	−0.7 (6)	Ir1—N2—C28—C21	−178.0 (2)
C8—C9—C10—C11	0.7 (6)	C20—C21—C28—N2	−1.4 (5)
C9—C10—C11—C6	−0.3 (5)	C22—C21—C28—N2	177.5 (3)
C9—C10—C11—Ir1	−179.5 (3)	C34—N3—C30—C31	−1.2 (5)
C7—C6—C11—C10	0.1 (5)	Ir1—N3—C30—C31	−176.5 (3)
C5—C6—C11—C10	−177.2 (3)	N3—C30—C31—C32	−0.1 (6)
C7—C6—C11—Ir1	179.3 (3)	C30—C31—C32—C33	0.4 (7)
C5—C6—C11—Ir1	2.1 (4)	C31—C32—C33—C34	0.6 (7)
C28—N2—C12—C19	2.6 (4)	C30—N3—C34—C33	2.2 (5)
Ir1—N2—C12—C19	179.9 (2)	Ir1—N3—C34—C33	178.0 (3)
C28—N2—C12—C13	−178.2 (3)	C30—N3—C34—C35	−176.1 (3)
Ir1—N2—C12—C13	−1.0 (3)	Ir1—N3—C34—C35	−0.3 (4)
N2—C12—C13—C18	−177.8 (3)	C32—C33—C34—N3	−2.0 (6)
C19—C12—C13—C18	1.2 (5)	C32—C33—C34—C35	176.1 (4)
N2—C12—C13—C14	1.9 (4)	N3—C34—C35—C40	176.4 (3)
C19—C12—C13—C14	−179.1 (3)	C33—C34—C35—C40	−1.8 (6)
C18—C13—C14—C15	1.5 (4)	N3—C34—C35—C36	−0.2 (5)
C12—C13—C14—C15	−178.2 (3)	C33—C34—C35—C36	−178.3 (4)
C18—C13—C14—Ir1	177.8 (2)	C40—C35—C36—C37	1.3 (5)
C12—C13—C14—Ir1	−1.9 (3)	C34—C35—C36—C37	177.9 (3)
C13—C14—C15—C16	−0.2 (5)	C40—C35—C36—Ir1	−176.1 (3)
Ir1—C14—C15—C16	−176.0 (3)	C34—C35—C36—Ir1	0.5 (4)
C14—C15—C16—C17	−0.9 (5)	C35—C36—C37—C38	−1.6 (5)
C15—C16—C17—C18	0.7 (5)	Ir1—C36—C37—C38	175.4 (3)
C16—C17—C18—C13	0.5 (5)	C36—C37—C38—C39	0.4 (6)
C14—C13—C18—C17	−1.7 (5)	C37—C38—C39—C40	1.1 (7)
C12—C13—C18—C17	178.0 (3)	C38—C39—C40—C35	−1.4 (7)
N2—C12—C19—C20	−1.7 (5)	C36—C35—C40—C39	0.2 (6)
C13—C12—C19—C20	179.3 (3)	C34—C35—C40—C39	−176.2 (4)
C12—C19—C20—C21	−0.8 (5)		

### Hydrogen-bond geometry (Å, º)

*Cg*1 and *Cg*2 are the centroids of the C22–C27 and N1/C1–C5 rings, respectively.

**Table:**

*D*—H···*A*	*D*—H	H···*A*	*D*···*A*	*D*—H···*A*
C29—H29*A*···*Cg*1^i^	0.98	2.89	3.589 (4)	136
C39—H39···*Cg*2^ii^	0.95	2.89	3.796 (5)	160

Symmetry codes: (i) −*x*, *y*−1/2, −*z*−1/2; (ii) −*x*+1, *y*−1/2, −*z*+1/2.
